# Endothelial Cpt1a Inhibits Neonatal Hyperoxia‐Induced Pulmonary Vascular Remodeling by Repressing Endothelial‐Mesenchymal Transition

**DOI:** 10.1002/advs.202415824

**Published:** 2025-01-12

**Authors:** Xiaoyun Li, Katy Hegarty, Fanjie Lin, Jason L. Chang, Amro Abdalla, Karthik Dhanabalan, Sergey O. Solomevich, Wenliang Song, Karim Roder, Chenrui Yao, Wenju Lu, Peter Carmeliet, Gaurav Choudhary, Phyllis A. Dennery, Hongwei Yao

**Affiliations:** ^1^ Department of Molecular Biology Cellular Biology, and Biochemistry Brown University Providence RI 02912 USA; ^2^ Providence VA Medical Center Providence RI 02908 USA; ^3^ Division of Cardiology Department of Medicine Warren Alpert Medical School of Brown University Providence RI 02903 USA; ^4^ College of Pharmacy Jinan University Guangzhou Guangdong 510632 China; ^5^ State Key Laboratory of Respiratory Disease Guangdong Key Laboratory of Vascular Disease National Clinical Research Center for Respiratory Disease Guangzhou Institute of Respiratory Health The First Affiliated Hospital of Guangzhou Medical University Guangzhou Guangdong 510120 China; ^6^ College of Arts & Sciences Boston University Boston MA 02215 USA; ^7^ Laboratory of Angiogenesis and Vascular Metabolism Department of Oncology and Leuven Cancer Institute KU Leuven VIB Center for Cancer Biology, VIB Leuven Brussels 3000 Belgium; ^8^ Center for Biotechnology Khalifa University Abu Dhabi 127788 UAE; ^9^ Department of Pediatrics Warren Alpert Medical School of Brown University Providence RI 02903 USA

**Keywords:** bronchopulmonary dysplasia, metabolic reprograming, nanoparticle‐mediated gene delivery, pulmonary hypertension, single‐cell RNA transcriptomics

## Abstract

Pulmonary hypertension (PH) increases the mortality of preterm infants with bronchopulmonary dysplasia (BPD). There are no curative therapies for this disease. Lung endothelial carnitine palmitoyltransferase 1a (Cpt1a), the rate‐limiting enzyme of the carnitine shuttle system, is reduced in a rodent model of BPD. It is unknown whether endothelial Cpt1a reduction causes pulmonary vascular (PV) remodeling. The latter can be the result of endothelial‐mesenchymal transition (EndoMT). Here, endothelial cell (EC)‐specific Cpt1a KO and WT mice (<12 h old) are exposed to hyperoxia (70% O_2_) for 14 days and allow them to recover in normoxia until postnatal day 28. Hyperoxia causes PH, which is aggravated in EC‐specific Cpt1a KO mice. Upregulating endothelial Cpt1a expression inhibits hyperoxia‐induced PV remodeling. Hyperoxia causes lung EndoMT, detected by immunofluorescence, scRNA‐sequencing, and EC lineage tracing, which is further increased in EC‐specific Cpt1a KO mice. Blocking EndoMT inhibits hyperoxia‐induced PV remodeling. Male mice under the same high oxygen conditions develop a higher degree of PH than females, which is associated with reduced endothelial Cpt1a expression. Conclusively, neonatal hyperoxia causes PH by decreasing endothelial Cpt1a expression and upregulating EndoMT. This provides a valuable strategy for developing targeted therapies by upregulating endothelial Cpt1a levels or inhibiting EndoMT to treat BPD‐associated PH.

## Graphical abstract

Endothelial‐mesenchymal transition (EndoMT) causes pulmonary vascular remodeling in a murine model of bronchopulmonary dysplasia‐associated pulmonary hypertension. Endothelial reduction of carnitine palmitoyltransferase 1a (Cpt1a) is identified as a novel mechanism of lung EndoMT. Endothelial Cpt1a deletion further increases, whereas nanoparticle‐mediated endothelial Cpt1a gene delivery inhibits pulmonary vascular remodeling and pulmonary hypertension in this mouse model.

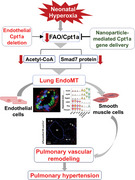

## 1 Introduction

Bronchopulmonary dysplasia (BPD), a chronic lung disease in premature infants, results from mechanical ventilation and hyperoxia amongst other factors.^[^ ^1^ ^]^ BPD affects 10 000–15 000 premature infants annually in the US. On average, each infant with BPD incurs a total cost of ≈$2 25 000 during the initial hospitalization.^[^ ^2^ ^]^ Approximately 30% of infants with moderate to severe BPD develop pulmonary hypertension.^[^ ^3^ ^]^ Male sex is an independent risk factor for this disease and associated pulmonary hypertension.^[^ ^4^ ^]^ Pulmonary vascular remodeling increases pulmonary vascular resistance leading to BPD‐associated pulmonary hypertension. There are no curative therapies for this disease, partially due to the lack of understanding of pathogenic mechanisms resulting in pulmonary hypertension. Current management is limited to relieving symptoms using pulmonary vasodilators and minimizing further lung vascular and alveolar insults.^[^ ^5^ ^]^ The unmet need is to develop novel targeted therapies to prevent and/or treat BPD‐associated pulmonary hypertension driven by a mechanistic understanding.

Pulmonary vascular remodeling is characterized by increased smooth muscle cell‐specific markers. This results from the proliferation and migration of vascular smooth muscle cells, or cellular trans‐differentiation from endothelial cells (ECs) (endothelial‐mesenchymal transition, EndoMT). The latter plays a pivotal role in mediating pulmonary vascular remodeling in idiopathic pulmonary hypertension.^[^ ^6^ ^]^ Hyperoxia in newborn mice and mechanical ventilation in preterm lambs result in pulmonary vascular remodeling, pulmonary hypertension, and right ventricular hypertrophy.^[^ ^7^ ^]^ This is associated with increased EndoMT, which is augmented in cultured lung ECs from male donors and in hyperoxia‐exposed male mice compared to those from females.^[^ ^7^, ^8^ ^]^ Although dysregulated metabolism occurs in newborns who develop pulmonary hypertension,^[^ ^9^ ^]^ no reports are showing that metabolic dysregulation contributes to the development of BPD‐associated pulmonary hypertension. Carnitine palmitoyltransferase 1a (Cpt1a) is the rate‐limiting enzyme of the carnitine shuttle responsible for transporting long‐chain fatty acids into mitochondria for β‐oxidation. We reported that neonatal hyperoxia causes a reduction of endothelial Cpt1a expression in mouse lungs.^[^ ^10^ ^]^ Whether endothelial Cpt1a reduction causes BPD‐associated pulmonary hypertension is unknown. We hypothesized that neonatal hyperoxia causes EndoMT by downregulating endothelial Cpt1a, thereby resulting in pulmonary vascular remodeling and pulmonary hypertension. To test this hypothesis, EC‐specific Cpt1a KO mice and WT littermates (<12 h old) were exposed to hyperoxia (70% O_2_) for 14 days and recovered in room air until postnatal day (pnd) 28. Dual immunofluorescence, reanalysis of publicly available single cell‐RNA transcriptomics datasets, and Rosa26‐tdTomato EC lineage tracing were carried out to assess the trajectory of and the mechanisms of lung EndoMT. In addition, sex differences in endothelial Cpt1a expression and enriched pathways regulating EndoMT were evaluated. Finally, therapeutic upregulation of Cpt1a using pharmacological treatments and nanoparticle‐mediated endothelial gene delivery as well as blockage of EndoMT were employed to determine their effects on neonatal hyperoxia‐induced pulmonary vascular remodeling and pulmonary hypertension.

## 2 Results

### 2.1 Neonatal Hyperoxia causes Pulmonary Vascular Remodeling, Right Ventricular Hypertrophy and Pulmonary Hypertension

**Figure 1:**
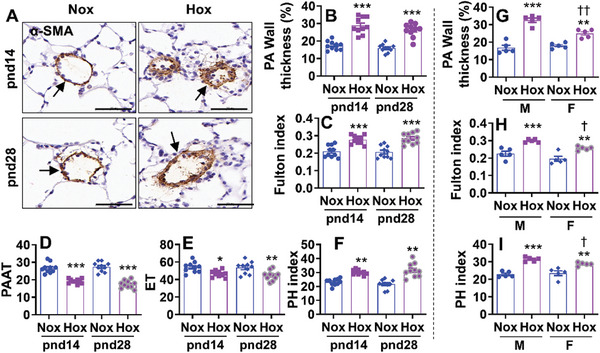
Neonatal hyperoxic exposure causes persistent pulmonary vascular remodeling, pulmonary hypertension, and right ventricular hypertrophy. C57BL/6J mice (<12 h old) were exposed to air (21% O_2_) or hyperoxia (70% O_2_) for 14 days. Some mice were allowed to recover in room air until pnd28. A) Immunohistochemistry was performed for α‐SMA in lung sections. Arrows denote vessels positive for α‐SMA. Bar size: 50 µm. B) Pulmonary arterial wall thickness was calculated in pulmonary arteries with <100 µm outer diameter based on α‐SMA staining (n = 10). C) Fulton index was calculated as the weight ratio of the right ventricle to the weight of the left ventricle plus septum) (n = 10). D–F) An echocardiogram was performed to record the hemodynamic parameters of PAAT and ET. The index of pulmonary hypertension (PH) was calculated with a regression formula: RVSP = 64.5‐83.5×PAAT/ET (n = 10). G–I) Sex differences in pulmonary arterial wall thickness, Fulton index, and PH index in mice exposed to hyperoxia as neonates at pnd14 (n = 5). Data are expressed as mean ± SEM. ^*^ *P* < 0.05, ^**^ *P* < 0.01, ^***^ *P* < 0.001 versus corresponding air group. ^†^ *P* < 0.05, ^††^ *P* < 0.01 versus hyperoxia in male mice. One‐way ANOVA followed by Tukey's post‐test was used for multiple comparisons.

To establish BPD‐associated pulmonary hypertension, C57BL/6J mice (<12 h old) were exposed to hyperoxia (70% O_2_) for 14 days. Some mice were allowed to recover in the air until pnd28. We first performed α‐SMA immunohistochemistry to determine pulmonary vascular remodeling by morphometric analysis in mice exposed to hyperoxia as neonates. As shown in **Figure** **1**A,B, neonatal hyperoxia increased pulmonary vascular muscularization as indicated by increased pulmonary arterial wall thickness. Right ventricular hypertrophy was evaluated by calculating the Fulton index as the weight ratio of the right ventricle to (left ventricle + septum). As shown in Figure 1C, the Fulton index was increased at both pnd14 and pnd28 mice exposed to hyperoxia as neonates. Echocardiography was performed to measure pulmonary artery acceleration time (PAAT) and ejection time (ET) for calculating an index of pulmonary hypertension. Neonatal hyperoxia significantly reduced both PAAT and ET and increased the index of pulmonary hypertension (Figure 1D–F). There were no significant changes in pulmonary arterial wall thickness, Fulton index, or the index of pulmonary hypertension between pnd14 and pnd28 after neonatal hyperoxia. Altogether, these data show that neonatal hyperoxia causes persistent pulmonary vascular remodeling, right ventricular hypertrophy, and pulmonary hypertension.

### 2.2 Male Mice are more Susceptible to Developing Pulmonary Vascular Remodeling and Pulmonary Hypertension

Male sex is an independent predictor for the development of BPD and associated morbidities.^[^ ^4^ ^]^ Therefore, we compared sex differences in neonatal hyperoxia‐induced pulmonary vascular and RV remodeling, and pulmonary hypertension. As shown in Figure 1G–I, neonatal hyperoxia increased pulmonary vascular wall thickness, Fulton index, and pulmonary hypertension in both male and female mice at pnd14. Compared to female mice, neonatal hyperoxia‐induced pulmonary vascular remodeling and pulmonary hypertension were further augmented in male mice at pnd14. Furthermore, right ventricular hypertrophy was also more apparent in male mice compared to female mice exposed to hyperoxia as neonates (Figure 1G–I). These results suggest that male mice under the same high oxygen conditions develop a higher degree of pulmonary hypertension than females.

### 2.3 Neonatal Hyperoxia causes EndoMT During the Development of Pulmonary Hypertension

**Figure 2:**
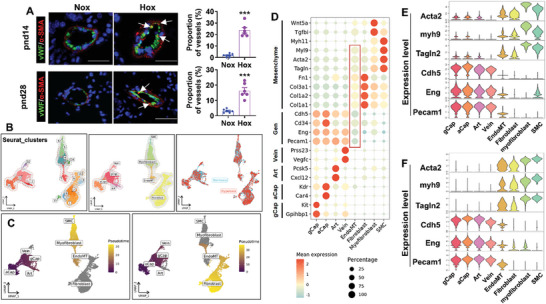
Neonatal hyperoxic exposure causes EndoMT in mouse lungs. A) C57BL/6J mice (<12 h old) were exposed to air (21% O_2_) or hyperoxia (70% O_2_) for 14 days. Some mice were allowed to recover in room air until pnd28. Immunofluorescence was performed for co‐staining of vWF and α‐SMA in the lungs of mice exposed to hyperoxia as neonates. Representative images are shown of mouse lungs at pnd14 and pnd28. Arrows denote cells co‐expressing α‐SMA and vWF. Quantification of lung vessels with ≤100 µm outer diameter exhibiting luminal co‐localization of α‐SMA/vWF at pnd14 and pnd28 when these mice were exposed to hyperoxia as newborns. Bar size: 20 µm. n = 6. A T‐test was used for comparison. B–E) Reanalysis of publicly available lung scRNA‐seq datasets from mice exposed to hyperoxia (85% O_2_) for 7 days^[^ ^11^ ^]^. B) UMAP plot representing the integration of lung ECs, fibroblasts, and smooth muscle cells (SMC) from air and hyperoxic groups. Single cells are colored by cluster identity. Sixteen clusters were detected. C) Trajectory analysis on UMAP plots suggests two putative relationships, namely “ECs‐EndoMT‐Fibroblasts” and “ECs‐EndoMT‐Myofibroblasts‐SMC”. Color represents cell localization along with pseudotemporal ordering. Grey clusters represent cells that are not aligned along the pseudotime axis. D) Dotplot shows expression of biomarkers of ECs and mesenchymal cells. The size of the dot indicates the proportion of cells within a cluster expressing the indicated gene, and the intensity of expression is indicated by the color legend. Gen, general. E) Violin plot shows expression of biomarkers of ECs (Pecam1, Eng, and Chd5) and mesenchymal cells (Acta2, Myl9, Tagln) in the cluster 7. E) Reanalysis of publicly available lung scRNA‐seq datasets from mice exposed to hyperoxia (85% O_2_) for 14 days^[^ ^11^ ^]^. F) Reanalysis of publicly available lung scRNA‐seq datasets from mice exposed to hyperoxia (95% O_2_) for 5 days (GSE211356)^[^ ^12^ ^]^. Data are expressed as mean ± SEM. ^***^ *P* < 0.001 versus the corresponding air group.

To determine whether neonatal hyperoxia causes EndoMT in the lung, we first performed immunofluorescence to detect co‐localization of an endothelial cell marker, von Willebrand Factor (vWF), and a mesenchymal cell biomarker α‐smooth muscle actin (α‐SMA) and focused on vessels with an outer diameter <100 µm. These small pulmonary vessels are the predominant site of remodeling in pulmonary hypertension. As shown in **Figure** **2**A, neonatal hyperoxia significantly increased the proportion of vessels co‐expressing α‐SMA (red) and vWF (green) in the lungs at both pnd14 and pnd28. There were ≈16%–25% of vessels with an outer diameter ≤100 µm co‐expressing α‐SMA/vWF at pnd14 and pnd28 after neonatal hyperoxia (Figure 2A). We next reanalyzed publicly available scRNA‐seq datasets in the lungs of mice exposed to hyperoxia (85% O_2_) for 7 and 14 days as neonates (GSE151974).^[^ ^11^ ^]^ We found a cluster of cells (cluster 7) expressing biomarkers for both ECs and mesenchymal cells including smooth muscle cells, fibroblasts, and myofibroblasts, suggesting EndoMT. The numbers of this cluster of cells were 249 and 128, which account for 18% and 9.8% of total ECs after 7 and 14 days of neonatal hyperoxia, respectively. Therefore, we evaluated the UMAP plots of EC subpopulations, mesenchymal cells (fibroblasts, myofibroblasts, and smooth muscle cells), and an intermediate cell state (EndoMT) (Figure 2B), trajectory assessment of EC transition into mesenchymal cells (Figure 2C) and expression of EC (Pecam1, Endoglin and Cdh5) and mesenchymal cell (Acta2, Myl9 and Tagln) biomarkers of these subpopulations (Figure 2D,E) after 7 days of neonatal hyperoxia. We further reanalyzed another scRNA‐seq dataset in the lung of mice exposed to hyperoxia (95% O_2_) for 5 days as neonates (GSE211356),^[^ ^12^ ^]^ and found a cluster of cells also expressing biomarkers for both ECs and mesenchymal cells at pnd7 (Figure 2F).

**Figure 3:**
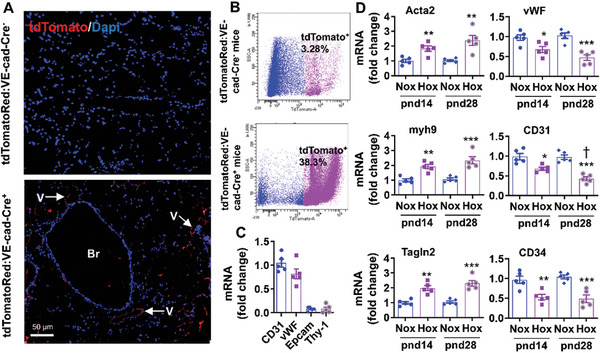
Lung EndoMT in EC tdTomato lineage traced mice exposed to hyperoxia as neonates. A) Lung tdTomato fluorescence in tdTomatoRed:VE‐cad‐Cre^−^ and tdTomatoRed:VE‐cad‐Cre^+^ mice under normoxia at pnd14. Br: Bronchiole; V: Vessel. B) Lung tdTomato^+^ cells were isolated by FACS from tdTomatoRed:VE‐cad‐Cre^−^ and tdTomatoRed:VE‐cad‐Cre^+^ mice under normoxia at pnd14. C) qRT‐PCR was carried out to measure gene expression of EC biomarkers CD31 and vWF, an epithelial cell biomarker Epcam, and a fibroblast biomarker Thy‐1 in lung tdTomato^+^ cells (n = 5). D) Lung tdTomato^+^ cells were isolated from tdTomatoRed:VE‐cad‐Cre^+^ mice exposed to air and hyperoxia for 14 days. Some mice were recovered in room air until pnd28. Expression of Acta2, myh9, Tagln2, vWF, CD31, and CD34 genes were evaluated by qRT‐PCR (n = 5). Data are expressed as mean ± SEM. ^*^ *P* < 0.05, ^**^ *P* < 0.01, ^***^ *P* < 0.001 versus corresponding air group. ^†^ *P* < 0.05 versus pnd14/hyperoxia. One‐way ANOVA followed by Tukey's post‐test was used for multiple comparisons.

EndoMT can be detected by immunofluorescence and scRNA‐seq only during a narrow time window when ECs express both endothelial and mesenchymal markers. Therefore, we employed cell‐lineage tracing for direct evidence of EndoMT in cells originating from ECs in mouse lungs after neonatal hyperoxia. To this effect, tdTomatoRed:VE‐cad‐Cre^+^ mice were generated (**Figure** **3**A). Lungs tdTomato^+^ ECs were isolated from these mice by FACS at pnd14. As shown in Figure 3B, tdTomato^+^ cells accounted for 38.3% and 3.28% of total lung cells in tdTomatoRed:VE‐cad‐Cre^+^ and tdTomatoRed:VE‐cad‐Cre^−^ mice, respectively. The tdTomato^+^ cells highly expressed EC biomarker genes (CD31 and vWF) but expressed epithelial cell (Epcam) and fibroblast biomarker (Thy‐1) to a low extent (Figure 3C). We then exposed tdTomatoRed:VE‐cad‐Cre^+^ mice to air or hyperoxia for 14 days as neonates. Some mice were recovered in room air until pnd28. Biomarkers of endothelial cells (vWF, CD31, and CD34) and mesenchymal cells (Acta2, myh9, and Tagln2) were measured using qRT‐PCR in lung tdTomato^+^ cells isolated from these mice. As shown in Figure 3D, neonatal hyperoxia significantly reduced the expression of EC biomarkers. In contrast, levels of Acta2, Myh9, and Tagln2 mRNAs were significantly increased in the tdTomato^+^ cells isolated from neonatal hyperoxia‐exposed mice at both pnd14 and pnd28 (Figure 3D). Altogether, these data demonstrate that neonatal hyperoxia causes lung EndoMT during the development of pulmonary hypertension.

### 2.4 Neonatal Hyperoxia Decreases Fatty Acid Oxidation (FAO) and Cpt1a Expression in Lung Endothelium

**Figure 4:**
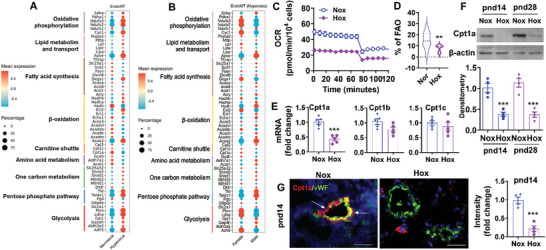
Hyperoxic exposure reduces FAO and Cpt1a expression in lung endothelium. A,B) Reanalysis of publicly available lung scRNA‐seq datasets from mice exposed to hyperoxia (85% O_2_) for 7 days^[^ ^11^ ^]^. Dotplot shows metabolic dysregulation in lung cells expressing both EC and mesenchymal cells between normoxia and hyperoxia (A), and between male and female mice (B). The size of the dot indicates the percentage of cells within a cluster expressing the indicated gene, and the intensity of expression is indicated by the color legend. C,D) Human fetal lung endothelial cells were exposed to hyperoxia (70% O_2_/5% CO_2_) or air (21% O_2_/5% CO_2_) for 24 h. FAO was then evaluated using the Seahorse XF24 Analyzer (n = 12). E) Expression of Cpt1a, Cpt1b, and Cpt1c genes was measured in these cells by qRT‐PCR (n = 5). F,G) C57BL/6J mice (<12 h old) were exposed to air (21% O_2_) or hyperoxia (70% O_2_) for 14 days. Some mice were allowed to recover in room air until pnd28. Lung ECs were isolated from these mice, and Cpt1a protein levels were detected by Western blot (F, n = 4). G) Immunofluorescence was performed for co‐staining of vWF and Cpt1a in the lung. Representative images are shown of mouse lungs at pnd14. Arrows denote cells co‐expressing α‐SMA and Cpt1a. Cpt1a fluorescence intensity was calculated by ImageJ and presented (n = 4). Bar size: 50 µm. Data are expressed as mean ± SEM. ^**^ *P* < 0.01, ^***^ *P* < 0.001 versus corresponding air group. T‐test (D, E, G) and one‐way ANOVA followed by Tukey's post‐test (F) were used for comparisons.

After reanalysis, existing scRNA‐seq data from GSE151974 showed that metabolism was dysregulated in cluster 7 and that the cells showed markers of both EC and mesenchymal cells after 7 days of neonatal hyperoxia (85% O_2_) (**Figure** **4**A). These metabolic changes included increased glycolysis (increased Ldha, Pkm, Gapdh, Gpi, and Slc2a1), an increased non‐oxidative branch of pentose phosphate pathway (increased TKT and Taldo), increased one‐carbon metabolism (increased Slc25a32, Shmt1, Shmt2, Mthfd1, and Mthfd2), reduced amino acid metabolism (Acat1, Aldh7a1, and Gcdh), dysregulated fatty acid synthesis (increased Acly, Acsl4 and Degs1, decreased Acaca, Fasn, Elovl6, Scd1, and Slc25a1), reduced carnitine shuttle (Cpt1a, Cpt1c and Cpt2), and dysregulated β‐oxidation (reduced Hadh, Echs1, Acadvl, Acads and Acadm, and increased Acaa2 and Eci2). In this cluster of cells, sex differences in metabolic gene expression were observed after neonatal exposure. This included reduced glycolysis (Pkm, Ldha, and Adh5), oxidative phosphorylation (Pdha1, Ndusf2, Ndusf3, and Cyc1), and carnitine shuttle (Cpt1a and Cpt1c) in male mice compared to female mice exposed to hyperoxia as neonates (Figure 4B).

A recent report shows that acetate, an endogenous metabolite of fatty acid β‐oxidation, controls EndoMT.^[^ ^13^ ^]^ We asked whether hyperoxia‐induced EndoMT is modulated by dysregulated FAO. We measured FAO by detecting mitochondrial fatty acid utilization using a Seahorse XF24 Analyzer according to the Seahorse XF Mito Fuel Flex Test protocol (Agilent Technologies) in cultured human fetal lung ECs exposed to hyperoxia^[^ ^10^ ^]^. As shown in Figure 4C,D, hyperoxic exposure (70%, 24 h) significantly reduced FAO in these cells. We then determined the gene expression of Cpt1 isoforms by qRT‐PCR in these cells and found that hyperoxia significantly decreased Cpt1a gene expression (Figure 4E). There were no changes in Cpt1b or Cpt1c gene expression in these cells exposed to hyperoxia (Figure 4E). Cpt1a protein levels were also reduced in lung ECs isolated from hyperoxia‐exposed mice at both pnd14 and pnd28 (Figure 4F). Immunofluorescence showed that endothelial Cpt1a expression was reduced at pnd14 in the lungs of mice exposed to hyperoxia as neonates (Figure 4G). These results demonstrate that neonatal hyperoxia reduces FAO and Cpt1a expression in lung endothelium.

### 2.5 Endothelial Cpt1a Reduction Contributes to Hyperoxia‐Induced EndoMT in the Lung

**Figure 5:**
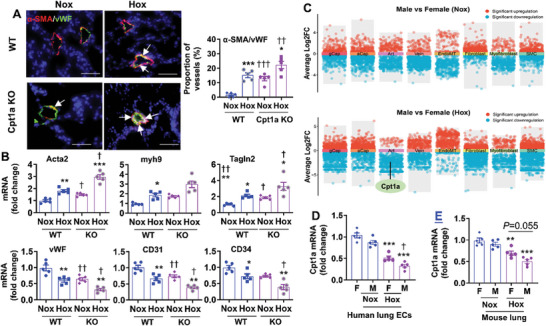
Endothelial Cpt1a deletion further augments neonatal hyperoxia‐induced EndoMT. A) EC‐specific Cpt1a KO mice (<12 h old) were exposed to air (21% O_2_) or hyperoxia (70% O_2_) for 14 days. Immunofluorescence was performed for co‐staining of vWF and α‐SMA in the lungs of mice exposed to hyperoxia as neonates. Representative images are shown of the colocalization of vWF and α‐SMA in mouse lungs. Arrows denote cells co‐expressing α‐SMA and vWF. Quantification of lung vessels with ≤100 µm outer diameter exhibiting luminal co‐localization of α‐SMA/vWF was presented (n = 5). Bar size: 20 µm. B) The tdTomatoRed:VE‐cad‐Cre:Cpt1aKO mice and WT littermates were exposed to air (21% O_2_) or hyperoxia (70% O_2_) for 14 days. Lung tdTomato^+^ cells were isolated from these mice. Expression of Acta2, myh9, Tagln2, vWF, CD31, and CD34 genes were evaluated by qRT‐PCR (n = 5). C) Reanalysis of publicly available lung scRNA‐seq datasets from mice exposed to normoxia (21% O_2_) or hyperoxia (85% O_2_) for 7 days^[^ ^11^ ^]^. Volcano plot shows significant upregulated (red) and downregulated (blue) gene expression in lung EC subpopulations, fibroblasts, myofibroblasts, and smooth muscle cells between male and female mice. D) Human fetal lung endothelial cells from male and female donors were exposed to hyperoxia (70% O_2_/5% CO_2_) or air (21% O_2_/5% CO_2_) for 24 h. E) C57BL/6J mice (<12 h old) were exposed to air (21% O_2_) or hyperoxia (70% O_2_) for 14 days. D,E) Cpt1a gene expression was measured by qRT‐PCR (n = 5). Data are expressed as mean ± SEM. ^*^ *P* < 0.05, ^**^ *P* < 0.01, ^***^ *P* < 0.001 versus corresponding normoxia. ^†^ *P* < 0.05, ^††^ *P* < 0.01, ^†††^ *P* < 0.01 versus corresponding WT group or female mice. One‐way ANOVA followed by Tukey's post‐test was used for multiple comparisons.

To determine whether reduced Cpt1a contributes to hyperoxia‐induced EndoMT, we exposed EC‐specific Cpt1a KO mice and their WT littermates to hyperoxia for 14 days and performed immunofluorescence to detect co‐localization of vWF and α‐SMA. As shown in **Figure** **5**A, neonatal hyperoxia increased the proportion of vessels co‐expressing vWF and α‐SMA in the lung of WT mice at pnd14. These changes were significantly increased in EC‐specific Cpt1a KO mice exposed to hyperoxia as neonates. Interestingly, under normoxia, endothelial Cpt1a deletion increased the proportion of lung vessels co‐expressing vWF and α‐SMA (Figure 5A). Additionally, endothelial Cpt1a deletion increased the expression of Acta2, Myh9, and Tagln2 genes in tdTomato^+^ EC isolated from tdTomato:VE‐cad‐cre^+^/Cpt1a^flox/flox^ mice under normoxia (Figure 5B). These effects were significantly enhanced at pnd14 after neonatal hyperoxia (Figure 5B). All these results demonstrate that endothelial Cpt1a reduction contributes to neonatal hyperoxia‐induced EndoMT in the lung.

### 2.6 Reduced Endothelial Cpt1a Expression in Males Compared to Females Exposed to Hyperoxia

We and others have shown that EndoMT is increased in hyperoxia‐exposed lung ECs isolated from male donors and in the lungs of hyperoxia‐exposed mice compared to those from females.^[^ ^7^, ^8^ ^]^ We asked whether sex differences of EndoMT were attributed to Cpt1a expression in response to hyperoxia. To this effect, we reanalyzed a scRNA‐seq dataset from GSE151974 in the lung of mice exposed to hyperoxia as neonates,^[^ ^11^ ^]^ and compared Cpt1a gene expression between male and female mice. As shown in Figure 5C, there were no differences in Cpt1a gene expression in the clusters of ECs, (myo)fibroblasts, or smooth muscle cells under normoxia. Under hyperoxia, the Cpt1a gene was significantly reduced in the cluster of lung arterial ECs from male mice compared to female mice (Figure 5C and Table S2, Supporting Information). We next exposed human fetal lung ECs from male and female donors at 18–24 weeks of gestation to hyperoxia (70%) for 24 h and measured Cpt1a gene expression by qRT‐PCR. As shown in Figure 5D, there were no sex differences in Cpt1a expression under normoxia. Compared to female cells, Cpt1a gene expression was decreased in male cells after hyperoxia. We finally evaluated lung Cpt1a gene expression at pnd14 in mice exposed to hyperoxia (70% O_2_) as neonates. As shown in Figure 5E, neonatal hyperoxia reduced lung Cpt1a gene expression in both sexes. There was a decreasing trend of lung Cpt1a gene expression in male mice compared to females after neonatal hyperoxia. Altogether, decreased Cpt1a expression may in part explain increased EndMT in male mice compared to female mice exposed to hyperoxia as neonates.

### 2.7 Cpt1a Maintains Smad7 Protein Expression and Replenishes Acetyl‐CoA in Lung ECs in Response to Hyperoxia

**Figure 6:**
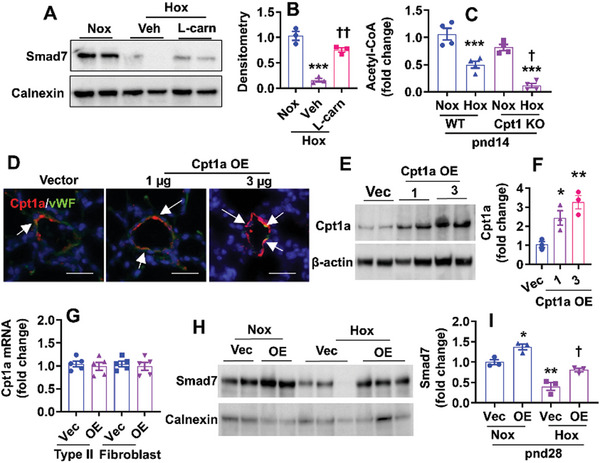
Cpt1a maintains Smad7 protein expression and replenishes acetyl‐CoA in response to hyperoxia. A,B) MFLM‐91U cells were exposed to 70% O_2_/5% CO_2_ for 24 h in the absence or presence of L‐carnitine (0.5 mm). Smad7 protein was detected by Western blot. N = 3 (C) EC‐specific Cpt1a KO mice and WT littermates (<12 h old) were exposed to 70% O_2_ for 14 days. Acetyl‐CoA levels were measured in lung ECs isolated from these mice (n = 4). D–I) C57BL/6J mice (<12 h old) were exposed to air (21% O_2_) or hyperoxia (70% O_2_) for 14 days and recovered in room air until pnd28. At pnd14 and pnd21, mixtures of nanoparticles mixed with 1 and 3 µg plasmid DNA expressing *Cpt1a* (Cpt1a OE) or empty vector under the control of human *CDH5* promoter were administered into mice via a retro‐orbital injection. Mice were euthanized at pnd28. D) Immunofluorescence of Cpt1a and vWF in the lung of mice administered with the mixtures of nanoparticles and plasmid DNA expressing *Cpt1a* (OE) or empty vector under normoxia. Bar size: 50 µm. E–G) Lung ECs (E, F, n = 3), epithelial cells, and fibroblasts (G, n = 5) were isolated from these mice under normoxia, and Cpt1a protein and gene expression were detected by Western blot and qRT‐PCR, respectively. H,I) Lung Smad7 protein levels were evaluated by Western blot at pnd28 in hyperoxia‐exposed mice administered with the mixtures of nanoparticles and 3 µg plasmid DNA expressing *Cpt1a* or empty vector (n = 3). ^*^ *P* < 0.05, ^**^ *P* < 0.01, ^***^ *P* < 0.001 versus corresponding normoxia, vector or normoxia/vector; ^†^ *P* < 0.05; ^††^ *P* < 0.01 versus hyperoxia/veh, WT/hyperoxia, or hyperoxia/vector. One‐way ANOVA followed by Tukey's post‐test was used for multiple comparisons.

We reported that hyperoxic exposure activates the TGF‐β/Smad pathway, leading to EndoMT.^[^ ^14^ ^]^ This is associated with reduced protein, but not mRNA expression of inhibitory Smad7 that negatively regulates TGF‐β signaling. A previous report shows that FAO‐derived acetyl‐CoA maintains the acetylation and stability of Smad7.^[^ ^15^ ^]^ Therefore, we evaluated Smad7 protein expression in hyperoxia (70% O_2_)‐exposed lung ECs treated with L‐carnitine (0.5 m, 12 h) which upregulates Cpt1a gene expression.^[^ ^16^ ^]^ L‐carnitine incubation restored hyperoxia‐induced reduction of Smad7 protein in cultured mouse fetal lung ECs (**Figure** **6**A,B). We then isolated lung ECs from EC‐specific Cpt1a KO and WT mice exposed to hyperoxia as neonates at pnd14, and measured acetyl‐CoA levels using a commercial kit. As shown in Figure 6C, neonatal hyperoxia significantly reduced acetyl‐CoA levels in lung ECs isolated from WT mice, which was further decreased in EC‐specific Cpt1a KO mice.

We recently reported that polylactide‐co‐glycolide (PLGA)/polyethylene glycol (PEG)‐based nanoparticles deliver plasmid DNA to lung vascular ECs in mice with a high efficiency.^[^ ^17^ ^]^ Thus, we encapsulated these nanoparticles with plasmid DNA expressing Cpt1a under the control of the human *CDH5* promoter or empty vector at a ratio of 1 µg plasmid DNA to 3 µL nanoparticles. Each C57BL/6J mouse received 1 and 3 µg of plasmid DNA at pnd14 and pnd21 after neonatal hyperoxia (70% O_2_) for 14 days.^[^ ^17^ ^]^ At pnd28, mice were euthanized and lung endothelial Cpt1a expression was evaluated by dual immunofluorescence. Under normoxia, endothelial Cpt1a expression was significantly increased in the lung of mice injected with nanoparticles mixed with plasmid DNA expressing Cpt1a compared to control (Figure 6D). We then evaluated Cpt1a expression in lung ECs, type II cells, and fibroblasts isolated from these mice. As shown in Figure 6E,G, nanoparticle‐mediated Cpt1a gene delivery significantly increased Cpt1a expression in lung ECs, but not in type II cells or fibroblasts. We next measured the Smad7 protein in the lungs of mice that had received nanoparticles mixed with plasmid DNA (3 µg) expressing the Cpt1a gene after neonatal hyperoxia. As shown in Figure 6H,I, neonatal hyperoxia reduced lung Smad7 protein level, which was attenuated by nanoparticle‐mediated endothelial Cpt1a overexpression.

**Figure 7:**
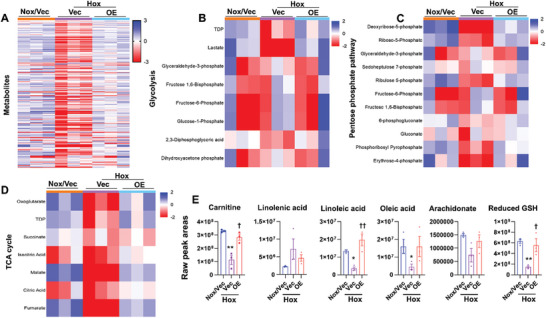
Endothelial Cpt1a overexpression maintains lung metabolism in mice exposed to hyperoxia as neonates. C57BL/6J mice (<12 h old) were exposed to air (21% O_2_) or hyperoxia (70% O_2_) for 14 days and recovered in room air until pnd28. At pnd14 and pnd21, mixtures of nanoparticles mixed with 3 µg plasmid DNA expressing *Cpt1a* (Cpt1a OE) or empty vector under the control of human *CDH5* promoter were administered into mice via a retro‐orbital injection. Mice were euthanized at pnd28. Lung metabolomics was performed. A) Heatmap illustrating metabolomic profiles among normoxia/vector (Nox/Vec), hyperoxia/vector (Hox/Vec), and hyperoxia/Cpt1a overexpression (Hox/OE) groups. Each row represents a metabolite, and each column corresponds to a sample. The color intensity reflects relative abundance [log(Z‐score)], highlighting differences in metabolite levels among these three groups. N = 3. B–D) Heatmap illustrating profiles of glycolysis, pentose phosphate pathway, and TCA cycle among three experimental groups. N = 3. E) Changes of carnitine, long‐chain fatty acids, and reduced glutathione among normoxia/vector (Nox/Vec), hyperoxia/vector (Hox/Vec), and hyperoxia/Cpt1a overexpression (Hox/OE) groups (n = 3). ^*^ *P* < 0.05, ^**^ *P* < 0.01 versus Nox/Vec; ^†^ *P* < 0.05; ^††^ *P* < 0.01 versus Hox/Vec. One‐way ANOVA followed by Tukey's post‐test was used for multiple comparisons.

We finally performed lung metabolomics at pnd28 in mice exposed to hyperoxia as neonates. As shown in **Figures** **7** and S1 (Supporting Information), lung levels of metabolites in glycolysis (glucose and lactate), the pentose phosphate pathway (erythrose‐4‐phosphate, deoxyribose‐5‐phosphate, ribose‐5‐phosphate, ribulose‐phosphate, 2‐deoxyglucose‐6‐phosphate, reduced glutathione), TCA cycle (fumarate and malate), amino acids (lysine, methionine, leucine, histidine, tryptophan, serine, asparagine, glutamine, and arginine), fatty acids (linoleic acid and oleic acid), and carnitine including acetyl carnitine, succinyl carnitine, and glutaral carnitine were reduced in hyperoxia‐exposed mice compared to normoxia. These effects were inhibited by nanoparticle‐mediated endothelial Cpt1a overexpression under hyperoxia. Altogether, Cpt1a maintains Smad7 protein expression, acetyl CoA levels, and the TCA cycle in response to hyperoxic exposure.

### 2.8 Endothelial Cpt1a Reduction Causes Neonatal Hyperoxia‐Induced Pulmonary Vascular Remodeling and Pulmonary Hypertension

**Figure 8:**
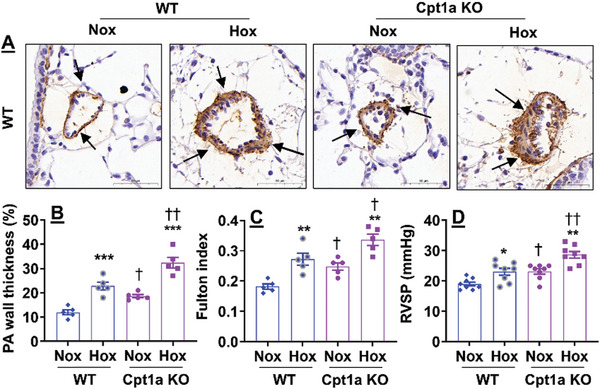
Endothelial Cpt1a deletion further augments neonatal hyperoxia‐induced pulmonary vascular remodeling and right ventricle hypertrophy. EC‐specific Cpt1a KO mice (<12 h old) were exposed to air (21% O_2_) or hyperoxia (70% O_2_) for 14 days. A) Immunohistochemistry was performed for α‐SMA in lung sections. Arrows denote vessels positive for α‐SMA. Bar size: 50 µm. B) Pulmonary arterial wall thickness was calculated in pulmonary arteries with <100 µm outer diameter based on α‐SMA staining (n = 5). C) Fulton index was evaluated as the weight ratio of the right ventricle to the weight of the left ventricle plus septum (n = 5). D) RVSP was recorded using an opened‐chest technique with a high‐fidelity pressure‐volume 1.0‐Fr catheter (n = 8). Data are expressed as mean ± SEM. ^*^ *P* < 0.05, ^**^ *P* < 0.01, ^***^ *P* < 0.001 versus corresponding air group. ^†^ *P* < 0.05, ^††^ *P* < 0.01 *vs* corresponding WT group. One‐way ANOVA followed by Tukey's post‐test was used for multiple comparisons.

To further determine the role of endothelial Cpt1a in modulating neonatal hyperoxia‐induced pulmonary hypertension, we evaluated pulmonary arterial wall thickness in vessels with an outer diameter <100 µm in EC‐specific Cpt1a KO mice and their WT littermates exposed to hyperoxia as neonates. As expected, neonatal hyperoxia increased pulmonary arterial wall thickness in WT mice at pnd28 (**Figure** **8**A,B). These effects were significantly enhanced in EC‐specific Cpt1a KO mice exposed to hyperoxia as neonates. Endothelial Cpt1a deletion also augmented the Fulton index after neonatal hyperoxia (Figure 8C). Finally, endothelial Cpt1a disruption further increased right ventricular systolic pressure (RVSP) in neonatal hyperoxia‐exposed mice at pnd28 (Figure 8D). Altogether, we show that endothelial Cpt1a deletion contributes to neonatal hyperoxia‐induced pulmonary vascular and right ventricular remodeling as well as pulmonary hypertension.

### 2.9 Pharmacological Upregulation of Cpt1a Inhibits Neonatal Hyperoxia‐Induced Pulmonary Vascular Remodeling and Pulmonary Hypertension

**Figure 9:**
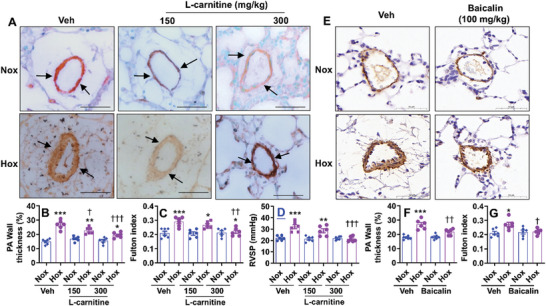
L‐carnitine and baicalin treatments inhibit neonatal hyperoxia‐induced pulmonary vascular remodeling and right ventricle hypertrophy. C57BL/6J mice (<12 h old) were exposed to air (21% O_2_) or hyperoxia (70% O_2_) for 14 days and recovered in room air until pnd28. L‐carnitine (150 and 300 mg kg^−1^) (A–D) and baicalin (100 mg kg^−1^) (E–G) were peritoneally injected into mice daily between pnd14 and pnd27. A, B, E, F) Immunohistochemistry was performed for α‐SMA in lung sections. Arrows denote vessels positive for α‐SMA. Bar size: 50 µm. Pulmonary arterial wall thickness was calculated in pulmonary arteries with <100 µm outer diameter based on α‐SMA staining (n = 6). C, G) The Fulton index was calculated as the weight ratio of the right ventricle to the left ventricle plus septum (n = 6). D) RVSP was recorded using an opened‐chest technique with a high‐fidelity pressure‐volume 1.0‐Fr catheter (n = 6). Data are expressed as mean ± SEM. ^*^ *P* < 0.05, ^**^ *P* < 0.01, ^***^ *P* < 0.001 versus corresponding air group; ^†^ *P* < 0.05 versus hyperoxia. One‐way ANOVA followed by Tukey's post‐test was used for multiple comparisons.

We previously reported that L‐carnitine upregulates lung endothelial Cpt1a expression.^[^ ^16^ ^]^ Here, we treated hyperoxia‐exposed mice with L‐carnitine (150 and 300 mg kg^−1^, i.p., daily) between pnd14 and pnd27 and determined pulmonary arterial wall thickness, Fulton index, and pulmonary hypertension at pnd28. As shown in **Figure** **9**A,B, L‐carnitine administration inhibited neonatal hyperoxia‐induced increases in pulmonary arterial wall thickness in a dose‐dependent manner. The Fulton index was significantly reduced in neonatal hyperoxia‐exposed mice treated with L‐carnitine (Figure 9C). L‐carnitine treatment also inhibited neonatal hyperoxia‐induced increases in RVSP (Figure 9D). Cpt1a can be directly bound and activated allosterically by baicalin.^[^ ^18^ ^]^ Baicalin incubation increases Cpt1a gene expression in lung ECs.^[^ ^16^ ^]^ Thus, we administered baicalin (100 mg kg^−1^, i.p.) between pnd14 and pnd27 and determined pulmonary arterial wall thickness and Fulton index at pnd28. As shown in Figure 9E–G, baicalin administration decreased neonatal hyperoxia‐induced increases in pulmonary arterial wall thickness and the Fulton index. Altogether, therapeutic upregulation of Cpt1a expression inhibits neonatal hyperoxia‐induced pulmonary vascular and right ventricular remodeling as well as pulmonary hypertension.

### 2.10 Nanoparticle‐Mediated Endothelial Cpt1a Gene Delivery Inhibits Neonatal Hyperoxia‐Induced EndoMT and Pulmonary Vascular Remodeling

**Figure 10:**
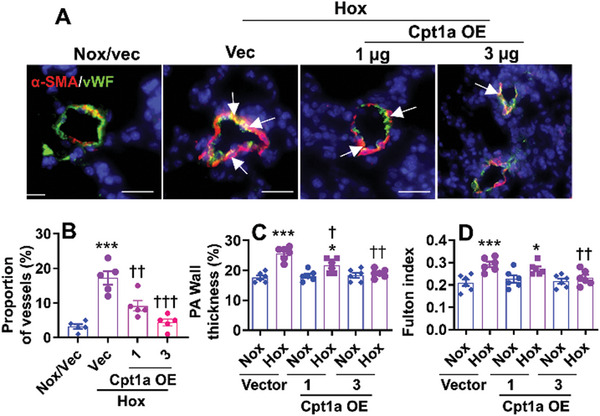
Nanoparticle‐mediated Cpt1a gene delivery inhibits neonatal hyperoxia‐induced EndoMT, pulmonary vascular remodeling, and right ventricle hypertrophy. C57BL/6J mice (<12 h old) were exposed to air (21% O_2_) or hyperoxia (70% O_2_) for 14 days and recovered in room air until pnd28. At pnd14 and pnd21, mixtures of nanoparticles and 1 and 3 µg plasmid DNA expressing *Cpt1a* (Cpt1a OE) or empty vector under the control of human *CDH5* promoter were administered into mice via a retro‐orbital injection. Mice were euthanized at pnd28. A, B) Immunofluorescence was performed for co‐staining of vWF and α‐SMA in the lungs of mice exposed to hyperoxia as neonates. Representative images are shown of mouse lungs, and arrows denote cells co‐expressing α‐SMA and vWF. Quantification of lung vessels with ≤100 µm outer diameter exhibiting luminal co‐localization of α‐SMA/vWF was presented (n = 5). Bar size: 50 µm. C) Immunohistochemistry was performed for α‐SMA in lung sections. Pulmonary arterial wall thickness was calculated in pulmonary arteries with <100 µm outer diameter based on α‐SMA staining (n = 6). D) The Fulton index was calculated as the weight ratio of the right ventricle to the left ventricle plus septum (n = 6). Data are expressed as mean ± SEM. N = 5–6. ^*^ *P* < 0.05, ^***^ *P* < 0.001 versus normoxia/vector (B) and corresponding normoxia group (C,D); ^†^ *P* < 0.05, ^††^ *P* < 0.01 versus vector/normoxia group. One‐way ANOVA followed by Tukey's post‐test was used for multiple comparisons.

Pharmacological treatments, including L‐carnitine, may have effects on other cells.^[^ ^19^ ^]^ Thus, we evaluated the effects of nanoparticle‐mediated endothelial Cpt1a overexpression on neonatal hyperoxia‐induced EndoMT and pulmonary vascular remodeling. As shown in **Figure** **10**A,B, nanoparticle‐mediated Cpt1a gene delivery inhibited the neonatal hyperoxia‐induced increase in the proportion of lung vessels co‐expressing vWF and α‐SMA. Furthermore, neonatal hyperoxia‐induced increases in pulmonary arterial wall thickness and Fulton index were reduced by nanoparticle‐mediated endothelial Cpt1a delivery (Figure 10C,D). This demonstrates that endothelial overexpression of Cpt1a inhibits neonatal hyperoxia‐induced pulmonary vascular remodeling.

### 2.11 Suppressing EndoMT Decreases Pulmonary Vascular Remodeling and Pulmonary Hypertension in both EC‐Specific Cpt1a KO and WT Mice

**Figure 11:**
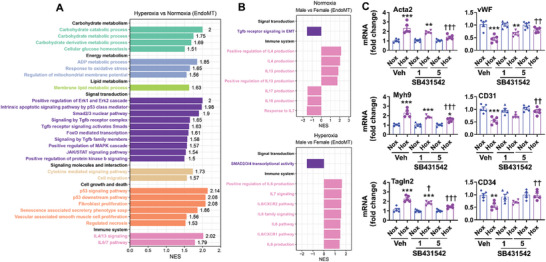
Enriched pathways in cells expression both EC and mesenchymal cell biomarkers after neonatal hyperoxia, and inhibiting TGFβ/Smad pathway represses hyperoxia‐induced EndoMT. A,B) Reanalysis of publicly available lung scRNA‐seq datasets from mice exposed to hyperoxia (85% O_2_) for 7 days^[^ ^11^ ^]^. Barplot shows the result of gene set enrichment analysis (GSEA) between hyperoxia‐impacted and normoxia (A), and between male and female mice (B) in cells expressing both EC and mesenchymal cell genes. All terms are significantly enriched (*P* value < 0.05) and normalized enrichment scores (NES) are shown. The length of each bar represents the magnitude of the NES, while the color codes for the class of pathways. C) Human fetal lung ECs were exposed to hyperoxia (70% O_2_/5% CO_2_) or air (21% O_2_/5% CO_2_) for 24 h in the presence or absence of SB431542 (1 and 5 µm). Gene expression was evaluated by qRT‐PCR (n = 5). Data are expressed as mean ± SEM. ^*^ *P* < 0.05, ^**^ *P* < 0.01, ^***^ *P* < 0.001 versus corresponding air group; ^†^ *P* < 0.05, ^††^ *P* < 0.01, ^†††^ *P* < 0.001 versus veh/hyperoxia. One‐way ANOVA followed by Tukey's post‐test was used for multiple comparisons.

The scRNA‐seq dataset from GSE151974 shows that TGF‐β receptor family members and the Smad2/3 nuclear pathway were enriched in lung cells expressing both EC and mesenchymal cell biomarkers after neonatal hyperoxia for 7 days (**Figure** **11**A). Other pathways, including the p53 pathway, inflammatory responses (ERK, JAK/STAT, IL‐4, and IL‐13), and cell migration and differentiation, were also enriched in cluster 7 of cells after neonatal hyperoxia (Figure 11A). Reduced TGF‐β/Smad2/3 pathway and increased IL‐6, IL‐7, and IL‐8 signals were observed in the cluster of cells expressing both EC and mesenchymal cell biomarkers from male mice compared to female mice exposed to hyperoxia as neonates (Figure 11B).

**Figure 12:**
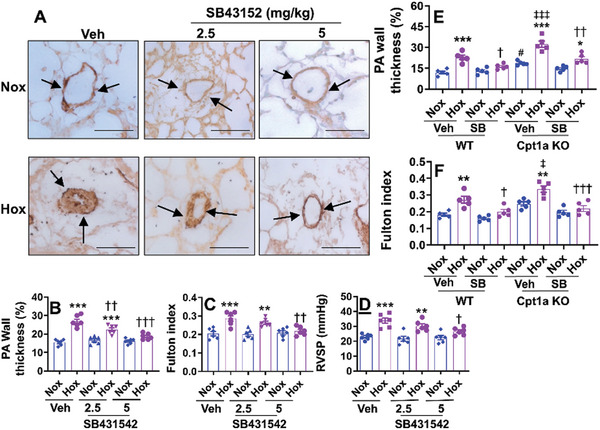
SB431542 treatment inhibits neonatal hyperoxia‐induced pulmonary vascular remodeling and right ventricle hypertrophy. C57BL/6J and EC‐Cpt1a KO mice (<12 h old) were exposed to air (21% O_2_) or hyperoxia (70% O_2_) for 14 days and recovered in room air until pnd28. SB431542 were peritoneally injected into C57BL/6J (2.5 and 5 mg kg^−1^) and EC‐Cpt1a KO (5 mg k^−1^g) mice daily between pnd14 and pnd27. A, B, E) Immunohistochemistry was performed for α‐SMA in lung sections. Arrows denote vessels positive for α‐SMA. Bar size: 50 µm. Pulmonary arterial wall thickness was calculated in pulmonary arteries with <100 µm outer diameter based on α‐SMA staining. N = 6 in panel B, and n = 5 in panel E. C, F) Fulton index was calculated as the weight ratio of the right ventricle to (left ventricle plus septum). N = 6 in panel C and n = 5 in panel F. D) RVSP was recorded using an opened‐chest technique with a high‐fidelity pressure‐volume 1.0‐Fr catheter (n = 5). Data are expressed as mean ± SEM. ^*^ *P* < 0.05, ^**^ *P* < 0.01, ^***^ *P* < 0.001 versus corresponding air group; ^†^ *P* < 0.05, ^††^ *P* < 0.01, ^†††^ *P* < 0.001 versus corresponding veh/hyperoxia (B–D) or WT/veh/hyperoxia (E, F); ^‡^ *P* < 0.05, ^‡‡‡^ *P* < 0.001 versus WT/veh/hyperoxia, ^#^ *P* < 0.05 versus WT/veh/Air. One‐way ANOVA followed by Tukey's post‐test was used for multiple comparisons.

We reported that hyperoxic exposure activates the TGFβ/Smad pathway and that inhibiting this pathway using SB431542 reduces hyperoxia‐induced EndoMT in cultured mouse lung ECs.^[^ ^14^ ^]^ Incubation of SB431542 also significantly reduced the expression of mesenchymal cell biomarkers (Acta1, myh9, and Tagln2) and restored the expression of endothelial biomarkers (vWF, CD31, and CD34) in cultured human fetal lung ECs exposed to hyperoxia (Figure 11C). We then evaluated whether SB431542 inhibits neonatal hyperoxia‐induced pulmonary vascular remodeling and pulmonary hypertension. As shown in **Figure** **12**A–D, administration of SB431542 (5 mg kg^−1^, ip, daily) between pnd14 and pnd27 in hyperoxia‐exposed mice significantly inhibited pulmonary vascular wall thickness, the Fulton index and RVSP at pnd28. Interestingly, SB431542 administration also inhibited neonatal hyperoxia‐induced pulmonary vascular wall thickness and the Fulton index in EC‐specific Cpt1a KO mice (Figure 12E,F). There were no significant differences in pulmonary arterial wall thickness and Fulton index between hyperoxia‐exposed EC‐specific Cpt1a KO and WT mice treated with SB431542 (Figure 12E,F). Altogether, these results suggest that endothelial Cpt1a deletion contributes to neonatal hyperoxia‐induced pulmonary vascular and right ventricular remodeling as well as pulmonary hypertension by upregulating EndoMT.

## 3 Discussion

Here, we show that neonatal hyperoxia causes EndoMT in the lung during the development of pulmonary hypertension. This is due to reduced Cpt1a expression and FAO in the endothelium in response to neonatal hyperoxia. Endothelial Cpt1a reduction contributes to, whereas Cpt1a upregulation represses, neonatal hyperoxia‐induced pulmonary vascular remodeling and pulmonary hypertension. Inhibiting EndoMT protects against neonatal hyperoxia‐induced pulmonary vascular remodeling in both EC‐specific Cpt1a KO mice (32.7% reduction) and their WT littermates (28.9% decrease). Therefore, neonatal hyperoxia causes Cpt1a reduction in lung endothelium, which results in EndoMT persistent pulmonary vascular remodeling and pulmonary hypertension.

The Cpt1a gene is upregulated by a high‐fat diet, which is associated with reduced methylation on the promoter and increased PPARα recruitment on the transcription start site.^[^ ^20^ ^]^ Neonatal hyperoxia causes persistent epigenetic changes including DNA methylation in the lung.^[^ ^21^ ^]^ This may lead to sustained Cpt1a reduction in mice exposed to hyperoxia as neonates. Further studies are warranted to determine the recruitment of PPARα on the Cpt1a promoter in response to hyperoxia. Endothelial Cpt1a deletion was further augmented, whereas nanoparticle‐mediated endothelial Cpt1a gene delivery inhibited, neonatal hyperoxia‐induced pulmonary vascular remodeling and right ventricular hypertrophy. In our model, neonatal hyperoxia decreased lung carnitine levels, and therapeutic upregulation of Cpt1a by L‐carnitine treatments also repressed neonatal hyperoxia‐induced pulmonary vascular remodeling. This agrees with previous studies showing that L‐carnitine prevents pulmonary vascular remodeling and right ventricular remodeling in rodent models of idiopathic pulmonary hypertension.^[^ ^22^ ^]^ We observed that endothelial Cpt1a deletion led to the spontaneous development of EndoMT and pulmonary vascular remodeling under normoxia. This highlights that hyperoxia‐induced Cpt1a reduction plays an important role in mediating EndoMT and subsequent pulmonary vascular remodeling. We reported that upregulating Cpt1a by L‐carnitine and baicalin prevents neonatal hyperoxia‐induced alveolar simplification by inhibiting EC apoptosis.^[^ ^10^, ^16^ ^]^ L‐carnitine reduces the duration of mechanical ventilation and the incidence of BPD in premature infants with respiratory distress syndrome.^[^ ^23^ ^]^ According to the guidelines of the American Thoracic Society and American Heart Association, management of pulmonary hypertension in BPD begins with aggressively treating the underlying lung disease.^[^ ^24^ ^]^ Therefore, Cpt1a upregulation (e.g., by L‐carnitine) that inhibits both alveolar injury and pulmonary vascular remodeling would be a significant and integrative approach to prevent the progression of BPD‐associated pulmonary hypertension.

Hyperoxia and mechanical ventilation activate the TGF‐β pathway, whereas TGF‐β neutralizing antibodies inhibit hyperoxic lung injury in rodents.^[^ ^25^ ^]^ This is corroborated by our scRNA‐seq data showing an activated TGF‐β/Smad pathway in cells expressing both EC and mesenchymal cell biomarkers after neonatal hyperoxia. We and others reported that a selective TGF‐β inhibitor SB431542 inhibits hyperoxia or TGF‐β‐induced EndoMT in vitro.^[^ ^7^, ^15^, ^26^ ^]^ This was corroborated in human fetal lung ECs as shown in the present study. Furthermore, treatment with SB431542 inhibits neonatal hyperoxia‐induced pulmonary vascular remodeling and right ventricular hypertrophy. This is consistent with findings showing that SB431542 treatment ameliorates *Schistosoma*‐induced pulmonary hypertension in adult mice,^[^ ^27^ ^]^ and suggests that inhibiting EndoMT ameliorates neonatal hyperoxia‐induced pulmonary hypertension. Interestingly, SB431542 treatment also inhibited pulmonary vascular remodeling and right ventricular hypertrophy in EC‐specific Cpt1a knockout mice exposed to hyperoxia as neonates. This indicates that Cpt1a deficiency results in EndoMT by activating the TGF‐β/Smad signal pathway during the development of neonatal hyperoxia‐induced pulmonary vascular remodeling. Previous studies have shown that FAO‐derived acetyl‐CoA maintains acetylation and stability of inhibitory Smad7, whereas Cpt1a or Cpt2 deletion decreases Smad7 levels and causes EndoMT.^[^ ^13^, ^15^ ^]^ Although Smad7 protein and acetyl‐CoA levels are modulated by Cpt1a in response to hyperoxia, further study is warranted to determine whether hyperoxia reduces Smad7 acetylation and stability through decreased FAO‐derived acetyl‐CoA, thereby leading to EndoMT. Further studies are required to determine whether impaired metabolism, including glycolysis, pentose phosphate pathway, TCA cycle, and amino acid metabolism, contributes to neonatal hyperoxia‐induced lung injury and vascular remodeling, as well as how Cpt1a overexpression restores this dysregulated metabolism.

We observed reduced Cpt2 gene, increased gene expression in glycolysis, and one‐carbon metabolism in lung cells expressing both EC and mesenchymal cell biomarkers after neonatal hyperoxia. This is in agreement with the findings that endothelial Cpt2 deficiency causes EndoMT in cardiac valves and the kidney and that glycolysis promotes EndoMT in the heart and aorta,^[^ ^15^, ^28^ ^]^ suggesting dysregulation of multiple metabolic pathways during neonatal hyperoxia‐induced EndoMT. The signaling protein p53 and inflammatory pathway were also enriched in lung cells undergoing EndoMT after neonatal hyperoxia. Previous studies have shown that nuclear p53 reverses EndoMT by promoting mesenchymal‐endothelial transition.^[^ ^29^ ^]^ Further studies are warranted to determine the role of p53 in modulating hyperoxia‐induced EndoMT and pulmonary vascular remodeling.

We noticed that lung endothelial Cpt1a was reduced in male mice compared to female mice exposed to hyperoxia as neonates. This may partially contribute to increased lung EndoMT and subsequent pulmonary vascular remodeling in the male sex.^[^ ^4^, ^7^, ^8^ ^]^ Interestingly, the TGF‐β/Smad2/3 pathway was downregulated in lung ECs undergoing EndoMT from male mice compared to female mice exposed to hyperoxia. In contrast, inflammatory responses, including IL‐6, IL‐7, IL‐8/CXCR1, and IL‐8/CXCR2 signals, were increased in lung cells from male mice versus female mice in response to hyperoxia. These findings suggest that sex differences of the TGF‐β/Smad2/3 pathway are not attributed to Cpt1a expression under hyperoxia. Palmitate upregulates expression of IL‐6, IL‐8, and ICAM‐1 expression in ECs, which leads to EndoMT.^[^ ^15^, ^30^ ^]^ Whether Cpt1a reduction contributes to sex differences in the inflammatory response resulting in EndoMT is unclear.

In conclusion, neonatal hyperoxia causes EndoMT and persistent pulmonary vascular remodeling. Inhibiting EndoMT attenuates neonatal hyperoxia‐induced pulmonary vascular remodeling and pulmonary hypertension. Furthermore, endothelial Cpt1a reduction contributes to neonatal hyperoxia‐induced EndoMT. Therapeutic upregulation of Cpt1a inhibits neonatal hyperoxia‐induced pulmonary vascular remodeling and right ventricular hypertrophy as well as pulmonary hypertension. This study provides a novel metabolic mechanism that explains neonatal hyperoxia‐induced pulmonary vascular remodeling and sex differences of this disease. This can serve to better guide the development of new therapies that upregulate endothelial Cpt1a or inhibit EndoMT to treat BPD‐associated pulmonary hypertension.

## 4 Experimental Section

### Hyperoxic Exposure

Newborn mice (<12 h old, male and female) along with their mothers were exposed to room air (21% O_2_) or hyperoxia (70% O_2_) for 14 days in an A‐chamber (BioSpherix, Redfield, NY). The dams were switched every 24 h between room air and hyperoxia to avoid injury. Some pups were allowed to recover in room air until pnd28. All animal experiments were reviewed and approved by the Institutional Animal Care and Use Committee of Brown University (IACUC: 21‐08‐0003).

Human fetal lung ECs (Cat#: 1001, ScienCell Research Laboratories) were maintained in an EC medium containing 5% FBS, EC growth supplement, and antibiotics at 37 °C in 5% CO_2_ and were used for experiments at passages 3–6. Mouse fetal lung EC lines (MFLM‐91U cells) were purchased from Seven Hills Bioreagents (Cincinnati, OH, USA), and grown in an Ultra‐Culture medium (BioWhittaker). Cells at 70%–80% confluence were exposed to hyperoxia (70% O_2_ and 5% CO_2_) or air (21% O_2_ and 5% CO_2_) for 24 h. Cells were treated with L‐carnitine (0.5 m) or SB431542 (1 and 5 µm) overnight.

### Generation of EC‐Specific Cpt1a Knockout and tdTomatoRed:VE‐cad‐Cre^+^ Mice

EC‐specific Cpt1a knockout mice were generated by crossing Cpt1a^lox/lox^ mice with C57BL/6J mice expressing the Cre recombinase transgene under control of the VE‐cadherin promoter (Stock#: 017968, Jackson Laboratory) as described in the reports.^[^ ^10^, ^16^, ^31^ ^]^ VE‐cadherin‐Cre mice were crossed with Rosa26‐tdTomato Red reporter mice (Stock#: 007909, Jackson Laboratory) to generate tdTomatoRed:VE‐cad‐Cre^+^ mice,^[^ ^32^ ^]^ where cells of endothelial origin were genetically labeled with red fluorescence. EC‐specific Cpt1a knockout mice were also crossed with Rosa26‐tdTomato Red reporter mice to generate tdTomatoRed:VE‐cad‐Cre:Cpt1aKO mice and WT littermates. PCR was performed to genotype and differentiate KO mice and WT littermates.

### Administration of L‐Carnitine and Baicalin in Mice

Neonatal hyperoxia (70% O_2_) for 14 days causes pulmonary vascular resistance and increases the Fulton index in mice.^[^ ^7^, ^33^ ^]^ L‐carnitine (150 and 300 mg kg^−1^) or baicalin (100 mg kg^−1^) were peritoneally injected into mice daily between pnd14 and pnd27 to determine their therapeutic effects on neonatal hyperoxia‐induced pulmonary hypertension. At pnd28, mice were sacrificed, and lungs were used for further analysis.

### Nanoparticle‐Mediated Endothelial Cpt1a Overexpression

The PEG‐b‐PLGA copolymer‐based nanoparticle formulated with polyethyleneimine was purchased from MountView Therapeutics, LLC (Deerfield, IL).^[^ ^34^ ^]^ The nanoparticles were mixed with plasmid DNA expressing *Cpt1a* or empty vector under the control of human *Cdh5* promoter at an optimized ratio of 1 µg plasmid DNA to 3 µL nanoparticles for 10 min at room temperature. Each C57BL/6J mouse received 1 and 3 µg of plasmid DNA via a retro‐orbital injection at pnd14 and pnd21 as described previously.^[^ ^17^ ^]^ Mice were sacrificed at pnd28.

### Echocardiographic Measurement

Under continuous isoflurane inhalation, mice were placed on a heated platform to maintain body temperature at 37 °C. Transthoracic echocardiography was carried out using a 40‐MHz linear‐array transducer (Vevo 2100; FIJIFILM VisualSonics, Toronto, Canada).^[^ ^35^ ^]^ PAAT and right ventricular ET were recorded and used to calculate the index of pulmonary hypertension with a regression formula: RVSP = 64.5–83.5×PAAT/ET.^[^ ^7^, ^33^ ^]^

### RVSP Measurement

Mice were anesthetized with isoflurane inhalation, and RVSP was measured using an opened‐chest technique.^[^ ^14^ ^]^ Briefly, a high‐fidelity pressure‐volume 1.0‐Fr catheter (PVR‐1030, Millar utilizing LabChart 8, AD Instruments, Colorado Springs, CO) was inserted into the left ventricular apex, and pressure measurements were recorded for 30–60 s. Subsequently, the same catheter was inserted into the apex of the right ventricle, and pressure measurements were recorded for another 30–60 s.

### Fulton Index Assessment

Following euthanasia with an injection of ketamine and xylazine, lungs and hearts were harvested and dissected. The right ventricle and left ventricle plus septum were weighed, and the Fulton index was calculated as the ratio of the right ventricle weight to the weight of the left ventricle plus septum).^[^ ^14^ ^]^

### Isolation of Lung Endothelial and Epithelial Cells and Fibroblasts

Mice were euthanized with an injection of ketamine and xylazine, and the lung was disassociated using a disassociation kit (Miltenyi Biotec, 130‐095‐927). Single‐cell suspension was prepared as described previously.^[^ ^36^ ^]^ Fluorescence‐activated cell sorting (FACS) was employed to sort tdTomato^+^ ECs in the lung from tdTomatoRed:VE‐cad‐Cre^+^ mice exposed to hyperoxia as neonates. Magnetic‐activated cell sorting (MACS) was utilized to isolate lung epithelial cells using an Epcam antibody (ThermoFisher Scientific, #12‐5791‐82).^[^ ^37^ ^]^ Lung fibroblasts were isolated by a crawl‐out method.^[^ ^38^ ^]^

### Immunohistochemistry

α‐SMA immunohistochemistry was carried out to calculate pulmonary arterial vessel thickness.^[^ ^35^ ^]^ Briefly, the lung was inflated with 1% low melt agarose at a pressure of 25 cm H_2_O and fixed with 4% neutral buffered paraformaldehyde. Fixed lung tissues were embedded in paraffin and sectioned into 5 µm sections using a rotary microtome. After antigen retrieval, the lung section was incubated with an α‐SMA monoclonal antibody (Table S1, Supporting Information) overnight at 4 °C, followed by a secondary antibody (Southern Biotech, Birmingham, AL). A Zeiss Axiovert 200 m Fluorescence Microscope was used to obtain digital images of the immunostaining. For each vessel, the external diameter and two wall thicknesses were measured along two different axes based on α‐SMA immunostaining. Wall thickness was calculated as (external–inner diameter)/external diameter × 100%, which was performed along two perpendicular axes.^[^ ^14^ ^]^ The two‐axis measurements were averaged together to determine the final wall thickness. A total of 5–6 vessels per mouse were utilized for calculating vascular wall thickness.

### Immunofluorescence

Lung sections were de‐paraffinized and subjected to heat‐mediated antigen retrieval in a citrate buffer solution (Vector Labs), then stained overnight at 4 °C with antibodies against vWF and α‐SMA (Table S1, Supporting Information). After incubation with secondary antibodies for 1 h at room temperature, sections were mounted in a hard‐set mounting medium containing DAPI (Vector Labs) and allowed to incubate overnight. Lung vessels with an outer diameter of ≤100 µm exhibiting luminal co‐localization of α‐SMA/vWF were quantified in a total of 3–4 vessels of 3–5 images per animal using a Zeiss Axiovert 200 M fluorescence microscope. The data were expressed as the average of the percentage of vessels positive for EndoMT per mouse. These experiments were carried out in a blinded manner.

### FAO Evaluation

Fatty acid utilization by mitochondria was evaluated by the Seahorse XF Analyzer (Seahorse Bioscience) following the Seahorse XF Mito Fuel Flex Test Assay.^[^ ^10^ ^]^ Briefly, oxygen consumption rate (OCR) was measured after injection of a specific Cpt1 inhibitor etomoxir (4 µm) and then a combination of a mitochondrial pyruvate carrier inhibitor UK5099 (2 µm) and a selective glutaminase inhibitor BPTES (3 µm). FAO measures reliance on a fatty acid pathway to maintain baseline respiration, which was calculated using the formula:

(1) $$\begin{array}{ccc} & & \mathrm{dependency}\;\left(\%\right)=\left(\mathrm{baseline}\;\mathrm{OCR}\;-\mathrm{etomoxir}\;\mathrm{OCR}\right)/\\ & & \left(\mathrm{baseline}\;\mathrm{OCR}-\mathrm{all}\;\mathrm{inhibitors}\;\mathrm{OCR}\right)\times 100 \end{array}$$

### Measurement of mRNA Levels by qRT‐PCR

Total RNA was extracted by the TRIzol reagent, and purified using the RNeasy miniprep kit (Qiagen, Valencia, CA). Levels of nucleic acids were detected using the NanoDrop One Microvolume UV–vis Spectrophotometer (Thermo Fisher Scientific, Waltham, MA). Reverse transcription was performed with a total of 400 nanograms of RNA using Taqman Reverse Transcription Reagents (Thermo Fisher Scientific). Real‐time PCR was carried out using 1 µL of cDNA by the 7300 Real‐Time PCR System (Applied Biosystems, Waltham, MA). All Taqman gene probes were purchased from Thermo Fisher Scientific (Table S1, Supporting Information). Gene expression was normalized to 18s rRNA levels. Relative RNA abundance was quantified by the comparative 2^−ΔΔCt^ method.

### Western Blot

Western blot was performed to detect protein levels in lysates which were prepared using 0.2 mL of RIPA buffer containing proteinase inhibitors. To disassociate the DNA, the lysates were passed 5 times through a 22‐gauge needle with the syringe. Samples were then kept on ice for 30 min to allow total cell lysis. A Pierce BCA Protein Assay kit (Thermo Scientific, Rockford, IL, USA) was used to measure protein levels. A total of 10–20 µg proteins were separated on a NuPAGE 4%–12% Bis‐Tris protein gel (Invitrogen), and electroblotted onto nitrocellulose membranes. After blocking with 5% BSA or nonfat dry milk for 1 h at room temperature, the membranes were then probed with 1:500–1:1000 diluted antibodies against Cpt1a, Smad7, and calnexin to determine their levels. Secondary antibodies with 1: 5000 dilutions in 5% BSA in PBS containing 0.1% Tween (v/v) 20 were incubated for 1 h, and the proteins detected by the ChemiDoc Touch Imaging System (BIO‐RAD) using the enhanced chemiluminescence method (Millipore). Antibody information is shown in Table S1 (Supporting Information). Equal loading of the samples was determined by quantification of proteins as well as by re‐probing membranes for the housekeeping control calnexin.

### Metabolomics Assay

Lung tissue samples (20 mg) were homogenized in 400 µL of cold 50% methanol containing 10 µL of QReSS isotopically labeled internal standards using a Polytron 2100 homogenizer (Kinematica, Switzerland) at 4 °C with the following settings: 20000 rpm, 4 × 10 s cycles, and a 120 s pause time. The homogenate was transferred to a 1.5 mL centrifuge tube, ensuring the tissue settled at the bottom of the tube without adhering to the walls. The samples were centrifuged at 15000 × g for 10 min at 4 °C to remove cellular debris. The resulting supernatant was filtered using cellulose acetate spin filters to remove particulate matter, dried, and stored at −80 °C until further processing.

On the day of analysis, dried samples were reconstituted in 400 µL of 50% methanol solution and passed through 0.2 µm micro‐centrifuge filter tubes. For quality control and method development, 50 µL of filtrates from each sample were pooled, while 100 µL of individual filtrates were transferred to LC vials (with 100 µL inserts) for analysis. Pooled quality control samples, double blanks (100% methanol), and blank internal standard samples (methanol spiked with QReSS standards) were included to ensure method reliability.

Metabolites were analyzed using a SCIEX Triple Quad 7500 LC‐MS/MS‐QTRAP system coupled with an ExionLC system. Chromatographic separation was conducted on a Kinetex F5 100 Å column (2.1 × 150 mm, 2.6 µm, Phenomenex, Torrance, CA, USA) at 30 °C, using binary mobile phases of water (A) and acetonitrile (B), both containing 0.1% formic acid (v/v). The gradient consisted of 0% B for 2.1 min, ramping to 95% B by 14 min, holding at 95% B for 2 min, and re‐equilibrating to 0% B from 16.1 to 20 min. The flow rate was maintained at 0.2 mL min^−1^, and samples were kept at 10 °C in the autosampler.

The optimized Scheduled MRM method was run in both positive and negative ionization modes, targeting over 800 metabolites using an established MRM list. Instrument settings included an ion source temperature of 350 °C, curtain gas at 40 psi and optimized GS1 and GS2 gas flows at 30 and 50 psi, respectively. Peaks were extracted and quantified using Analyst 3.1 software (AB Sciex, Framingham, MA, USA). Internal standards were used to normalize metabolite levels, and metabolite abundance was further normalized to tissue weight. Downstream analyses were performed using Metaboanalyst (v. 6.0) and GraphPad Prism (v. 10.0). Quality control included filtering metabolites with poor peak quality, ensuring unique and high‐quality features were used for the final analysis.

### Acetyl‐CoA Measurement

Acetyl‐CoA levels were measured using a commercial kit according to the manufacturer's instructions (Abcam, Cat#A319654).

### Reanalysis of Publicly Available scRNA‐seq Datasets

scRNA‐seq sequencing datasets were utilized that were publicly available from previously published studies with GEO accession numbers GSE151974 and GSE211356.^[^ ^11^, ^12^ ^]^ All main processing steps were performed with Seurat v.4.1.1.^[^ ^39^ ^]^

For the GSE151974 dataset, the preliminary quality control, integration, and clustering were performed as described previously.^[^ ^11^ ^]^ Annotated ECs, fibroblasts, myofibroblasts, and smooth muscle cells (SMC) were extracted as a subset and split each group (Hyperoxia and normoxia) at pnd7 into a separate Seurat object. Data were normalized and scaled using the SCTransform method with glmGamPoi^[^ ^40^ ^]^ and regressing out the effects of cell cycle score (G2M.Score, S.Score) and percentage fraction of mitochondria RNA and number of unique molecular identifier (UMI) per cell. For integration of separate Seurat objects, normalized values from SCTransform and the top 3000 variable genes as anchors were applied for canonical correlation analysis (CCA) using the SelectIntegrationFeatures(), PrepSCTIntegration(), FindIntegrationAnchors(), and IntegrateData() functions with default options. Linear dimension reduction was performed using principal component analysis (PCA), and the number of PCA dimensions was evaluated and selected based on the assessment of an ElbowPlot (Selected dims = 1:40). The top 40 statistically significant PCs were used in the FindNeighbors() function. Data were clustered using the Louvain‐graph‐based algorithm implemented in the FindClusters() function and cluster resolution was chosen in accordance with evaluation by the clustree v0.5.1^[^ ^41^ ^]^ (Selected resolution = 0.5). The uniform manifold approximation and projection (UMAP) reduction algorithm was performed to embed the clusters into 2D coordinates. Subsequently, canonical marker genes were used to identify a cluster of ECs, EndoMT, and mesenchymal cells: gCap: *Gpihbp1* and *Kit*; aCap: *Car4* and *Kdr*; Art: *Cxcl12* and *Pcsk5*; Vein: *Vegfc* and *Prss23*; general EC: *Pecam1*, *Eng*, *Cd34* and *Cdh5*; Fibroblast: *Col1a1*, *Col1a2*, *Col3a1* and *Fn1*; Myofibroblast: *Tgfbi* and *Wnt5a*; SMC: *Tagln*, *Acta2*, *Myl9* and *Myh11*.

CellRanger output matrices (10× Genomics) for each sample of the GSE211356 dataset were loaded to create Seurat objects using the Read10×() function. For quality control, cells with less than 200 genes, greater than three median absolute deviations above the median, and greater than 5% mitochondrial reads were removed. Potential doublets were identified using the scds v1.20.0,^[^ ^42^ ^]^ and removed. DecontX v1.0.0^[^ ^43^ ^]^ was applied to estimate and filter a contamination factor of 0.2 in individual cells. Each unique sample was then split into a separate Seurat object. Data were normalized and scaled using the SCTransform method with glmGamPoi and regressing out the effects of percentage fraction of mitochondria and number of UMI per cell. Integration and downstream analysis were the same as above, except for selecting a clustering resolution of 0.3. Subsequently, canonical marker genes were used to identify epithelial, endothelial, immune, and mesenchymal cells: Epcam, Pecam1, Col1a1, and Ptprc. ECs and mesenchymal cells were then extracted as a subset and each sample was splitted into a separate Seurat object for re‐processing, integrating, and clustering (resolution = 0.7). Specific clusters of ECs, EndoMT, and mesenchymal population were finally identified (markers are the same as above).

For intergroup gene expression differences, PrepSCTFindMarkers() and FindMarkers() functions were employed to identify variation between specified clusters using the Wilcox algorithm (Parameters: logfc.threshold = 0, min.cells.group = 1). A list of gene sets comprising all GO biological process terms, KEGG pathways, Reactome pathways, PID pathways, BioCarta pathways, and WikiPathways was acquired from the Molecular Signatures Database (v7)^[^ ^44^ ^]^ to identify major transitions associated with hyperoxia‐ and sex‐specific conditions of EndoMT on the fold‐change‐ranked list of genes using fgsea v1.30.0 (Parameters: minSize = 5, maxSize = 500, nperm = 10 000). Significantly enriched gene sets were defined with the criteria of *P* value < 0.05, and the normalized enrichment score (NES) was used to assess whether these gene sets were associated with upregulated or downregulated genes in hyperoxia versus normoxia or male versus female mice. Trajectory analysis was performed on the UMAP coordinates using the Slingshot v2.12.0^[^ ^45^ ^]^ with ECs (gCap, aCap, Art, and Vein clusters) as an assigned starting point of cell development trajectory without assigned end points to infer the pseudo‐temporal order of EndoMT.

All figures as part of the scRNA‐seq analysis were generated with ggplot2 v3.5.0, and all analyses were run in R v4.4.0 and RStudio v2023.12.0.

### Statistical Analysis

The results were expressed as mean ± standard error of the mean (SEM). Statistical analyses were performed using GraphPad Prism 10 software. The statistical significance of the differences was evaluated by using one‐way ANOVA followed by Tukey's post‐test to specifically compare indicated groups during multiple comparisons. The t‐test was used to detect the statistical significance of the differences between the means of the two groups after checking the normality of the data. For in vitro studies, statistical analysis was performed on at least three biological replicates. For in vivo studies, the sample size was at least 5 animals per group. *P* values*>*0.05 were considered insignificant, while statistical significance was considered at a *P* value of <0.05. The following symbols were used to indicate the level of significance: ^*^, ^†^, ^‡^, ^#^ *P* for *P < *0.05, ^**^, ^††^, ^‡‡^, ^##^ *P* for *P < *0.01, ^***^, ^†††^, ^‡‡‡^, ^###^ *P* for *P < *0.001.

### Code Availability

Analysis associated with the scRNA‐seq datasets used published R packages and custom functions, which are available at the GitHub repository (https://github.com/LWJlab/scRNA‐seq).

## Conflict of Interest

The authors declare no conflict of interest.

## Author Contributions

H.Y. designed the study, conducted experiments, acquired and analyzed data, drafted the initial manuscript, and revised the manuscript. X.L., K.H., J.L.C., S.O.S., W.S., and G.C. performed experiments and data analysis. K.M. performed a molecular clone of Cpt1a plasmids. F.L., C.Y., and W.L. reanalyzed publicly available scRNA‐seq datasets. P.C. generated Cpt1a^flox/flox^ mice and edited the manuscript. P.A.D. assisted with the experimental design and edited the manuscript. All authors approved the final manuscript as submitted.

## Supporting information



Supporting Information

Supplemental Table 2

## Data Availability

The data that support the findings of this study are available in the supplementary material of this article.
