# Supplementary material for: Endothelial Cpt1a Inhibits Neonatal Hyperoxia‐Induced Pulmonary Vascular Remodeling by Repressing Endothelial‐Mesenchymal Transition
Source: Adv Sci (Weinh). 2025 Jan 12;12(11):2415824. doi: 10.1002/advs.202415824 (PMC11923872; doi:10.1002/advs.202415824)
Supplement: Supplementary file 1 — Supporting Information [file ADVS-12-2415824-s002.docx]

Supporting Information

**Endothelial Cpt1a Inhibits** **Neonatal Hyperoxia-Induced Pulmonary Vascular Remodeling by Repressing Endothelial-Mesenchymal Transition**

*Xiaoyun Li, Katy Hegarty, Fanjie Lin, Jason L. Chang, Amro Abdalla, Karthik Dhanabalan, Sergey O. Solomevich, Wenliang Song, Karim Roder, Chenrui Yao, Wenju Lu, Peter Carmeliet, Gaurav Choudhary, Phyllis A. Dennery, Hongwei Yao^*^*

**
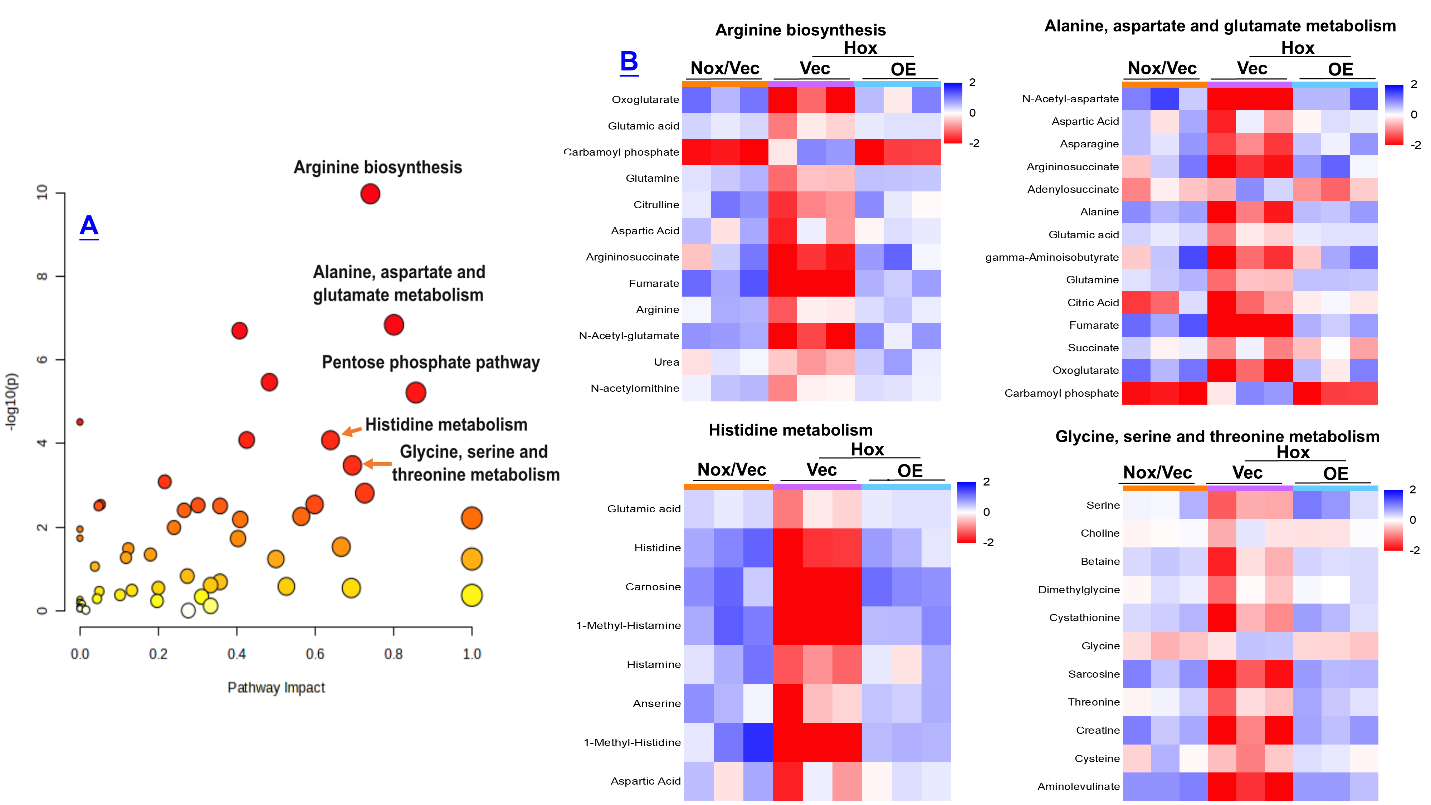
**

**Figure S1.** Endothelial Cpt1a overexpression maintains lung metabolism in mice exposed to hyperoxia as neonates. C57BL/6J mice (<12 h old) were exposed to air (21% O_2_) or hyperoxia (70% O_2_) for 14 days and recovered in room air until pnd28. At pnd14 and pnd21, mixtures of nanoparticles mixed with 3 µg plasmid DNA expressing *Cpt1a* (Cpt1a OE) or empty vector under the control of human *CDH5* promoter were administered into mice via a retro-orbital injection. Mice were euthanized at pnd28. Lung metabolomics was performed. (A) Overview of metabolic pathway analysis with *P*<0.05 and impact>0.5. Five pathways are shown with significant p-values and high impact scores. (B) Heatmap illustrating amino acid metabolism among normoxia/vector (Nox/Vec), hyperoxia/vector (Hox/Vec), and hyperoxia/Cpt1a overexpression (Hox/OE) groups. Each row represents a metabolite, and each column corresponds to a sample. The color intensity reflects relative abundance [log(Z-score)], highlighting differences in metabolite levels among these three groups.

**Table S1.** Antibodies and probes used in this study

| Name | Dilution | Cat# | Company |
| --- | --- | --- | --- |
| Antibodies | | | |
| α-smooth muscle actin (α-SMA) | 1:1000 for WB, 1:100 for immunostaining | ab21027 | Abcam |
| Cpt1a | 1:1000 (WB), 1:100 (IF) | ab128568 | Abcam |
| β-actin | 1:1000 | ab8227 | Abcam |
| Smad7 | 1:1000 | ab62533 | Abcam |
| von Willebrand factor (vWF) | 1:1000 (WB), 1:100 (IF) | ab201336 | Abcam |
| Calnexin | 1:10000 | ADI-SPA-860-F | Enzo Life Sciences |
| PCR probes | | | |
| Acta2 |  | [Mm00483012_s1](https://www.thermofisher.com/taqman-gene-expression/product/Mm00483012_s1?CID=&ICID=&subtype=) | ThermoFisher Scientific |
| Myh9 |  | Mm01197036_m1 | ThermoFisher Scientific |
| Tagln2 |  | Mm00724260_g1 | ThermoFisher Scientific |
| vWF |  | Mm00550376_m1 | ThermoFisher Scientific |
| CD31 |  | Mm01242576_m1 | ThermoFisher Scientific |
| CD34 |  | Mm00519283_m1 | ThermoFisher Scientific |
| Epcam |  | Mm00493214_m1 | ThermoFisher Scientific |
| Thy-1 |  | Mm00493681_m1 | ThermoFisher Scientific |
| Acta2 |  | Hs00426835_g1 | ThermoFisher Scientific |
| Myh9 |  | Hs00159522_m1 | ThermoFisher Scientific |
| Tagln2 |  | Hs00761239_s1 | ThermoFisher Scientific |
| vWF |  | Hs01109446_m1 | ThermoFisher Scientific |
| CD31 |  | Hs01065279_m1 | ThermoFisher Scientific |
| CD34 |  | Hs02576480_m1 | ThermoFisher Scientific |
| Cpt1a |  | Hs00912671_m1 | ThermoFisher Scientific |
| Cpt1b |  | Hs03046298_s1 | ThermoFisher Scientific |
| Cpt1c |  | Hs00380581_m1 | ThermoFisher Scientific |

WB: Western blot; IF: immunofluorescence
